# Modeling Control Strategies of Respiratory Pathogens

**DOI:** 10.3201/eid1108.040449

**Published:** 2005-08

**Authors:** Babak Pourbohloul, Lauren Ancel Meyers, Danuta M. Skowronski, Mel Krajden, David M. Patrick, Robert C. Brunham

**Affiliations:** * University of British Columbia Centre for Disease Control, Vancouver, British Columbia, Canada;; † University of Texas at Austin, Austin, Texas, USA;; ‡ Santa Fe Institute, Santa Fe, New Mexico, USA

**Keywords:** epidemic, immunization, infection control, mathematical model, outbreak, quarantine, Disease Transmission, Respiratory-borne infection, bioterrorist attack, emerging infectious disease

## Abstract

Effectively controlling infectious diseases requires quantitative comparisons of quarantine, infection control precautions, case identification and isolation, and immunization interventions. We used contact network epidemiology to predict the effect of various control policies for a mildly contagious disease, such as severe acute respiratory syndrome, and a moderately contagious disease, such as smallpox. The success of an intervention depends on the transmissibility of the disease and the contact pattern between persons within a community. The model predicts that use of face masks and general vaccination will only moderately affect the spread of mildly contagious diseases. In contrast, quarantine and ring vaccination can prevent the spread of a wide spectrum of diseases. Contact network epidemiology can provide valuable quantitative input to public health decisionmaking, even before a pathogen is well characterized.

## Abstract 2

Contact network epidemiology can provide quantitative input even before pathogen is fully characterized.

Public concern regarding emerging infectious diseases is on the rise. The 21st century began with the emergence or reemergence of zoonotic diseases like severe acute respiratory syndrome (SARS) (*1*), avian influenza (*2*), monkeypox infection (*3*), West Nile virus disease (*4*), mad cow disease (*5*), anthrax due to bioterrorist attacks (*6*), and unusual influenza epidemics (*7*). In addition to these new threats, public health officials face a large number of disease outbreaks every year in hospitals, schools, and other small communities. While development of vaccines and diagnostic tools proceeds at an unprecedented pace, development of tools for determining optimal intervention strategies lags behind.

In response to this problem, we have found that mathematical models of disease transmission can be used to evaluate and optimize control strategies. Such quantitative predictions can be empirically tested through randomized comparative trials, and mathematical models increasingly contribute to public health decisions regarding policy and intervention (*8**–**13*).

We use contact network epidemiology to compare intervention strategies for airborne ^2^ infectious diseases, including emerging diseases such as SARS, for which epidemiologic data are limited. These methods are based on explicit mathematical models of the heterogeneous patterns of interpersonal contacts that underlie disease transmission in a community, be it a hospital, school, or city (*12**–**21*). This approach differs from fully mixed compartmental models that assume that each person can infect every other person with equal probability (*8*). Some compartmental models have been modified to include population heterogeneity and have provided insights into the long-term effects of intervention strategies (*8**–**11*). For communities with extensive heterogeneity in contact patterns, however, network models more explicitly capture patterns of disease transmission and thus enable more accurate and detailed predictions of the effect of control measures on the magnitude and distributions of outbreaks.

## Methods

Contact network models capture and estimate interpersonal contacts that lead to disease transmission within a community (*22*). Contacts can take place within households, schools, workplaces, hospitals, and other public venues. Each person in a community is represented as a vertex in the network, and each contact between 2 people is represented as an edge connecting the vertexes. The number of edges emanating from a vertex is the degree of that vertex. This quantity indicates the number of contacts who potentially transmit disease to or acquire disease from a person. The variation of degree across the entire network, i.e., the degree distribution, is fundamental to determining the probability for spread of disease through a network of contacts. Given the degree distribution of the contact network, one can analytically predict the fate of an outbreak.

Contact network epidemiology allows us to assess the vulnerability of a population to an infectious disease on the basis of the structure of the network (its degree distribution) and on the average transmissibility (*T*) of the disease (*12**,**13*). *T* is the average probability that transmission will occur from an infected person (vertex) to an uninfected person. This parameter summarizes multiple aspects of transmissibility, including the contact intensity between persons, duration of infectiousness, and the host's susceptibility to the infectious pathogen (*12**,**13*).

### Contact Network Parameter Estimation

We built an urban contact network model with 2,000 households with an average household size of 2.6 (5,154 persons) based on demographic information for the Greater Vancouver Regional District, British Columbia, Canada. We used publicly available data from sources such as Statistics Canada to estimate the distribution of ages, household sizes, school and classroom sizes, hospital occupancy, workplaces, and public spaces (*23**–**27*).

**Figure 1:**
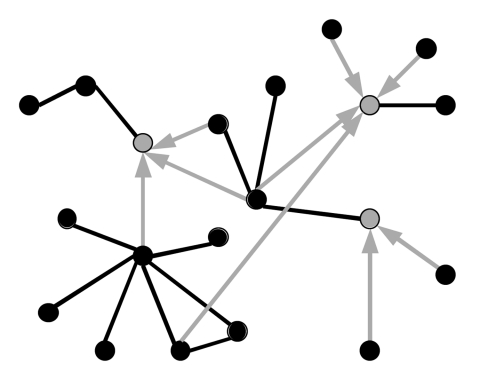
Schematic diagram of a directed network. Each black vertex represents a member of the general population; gray vertexes represent healthcare workers.

Most of the edges in the network are undirected, meaning that transmission may occur in either direction (black edges in Figure 1). For example, 2 persons living in the same household will have equal opportunities to infect each other. The remaining edges are directed, meaning that a person may infect another person but the converse is not true (gray edges in Figure 1). For example, suppose person A is healthy and has no reason to go to the hospital until he or she is infected with SARS. At that point, person A will likely come into contact with and potentially spread SARS to caregivers at the hospital. In contrast, if a caregiver at the hospital acquired SARS while person A remained healthy in the community, then no opportunity would exist for transmission in the opposite direction. To model the directional flow of infected patients into a hospital, we include directed edges from persons in the population at large to caregivers in the hospital.

In an urban setting, not all encounters are equally likely to lead to disease transmission. We capture this heterogeneity in 2 ways. First, in the simulated urban network, the probability of a contact between 2 persons depends on the location and nature of their overlapping daily activities. For example, persons in the same household are connected to each other with probability 1, while persons who encounter each other in a public space are connected to each other with a probability from 0.003 to 0.300. Second, after these connections are determined, we assign a distinct transmissibility, *T_ij_*, for each pair of connected persons *i* and *j*, that depends on the nature of their contact. For a given disease, the distribution of transmissibilities is based on empiric estimates for the diversity in infectious periods and the per day probability of transmission between persons who come into contact with each other. For more details, please refer to the Appendix.

### Modeling Control Strategies

In any given network exists a critical transmissibility value, *T_c_*, which indicates whether a large-scale epidemic is probable. Any disease with average transmissibility <*T_c_* cannot cause sustained transmission within a population and will thus be limited to small outbreaks. Such diseases die out because of the probabilistic nature of transmission before the disease has a chance to spread to the population at large. In this case, we can mathematically predict the expected size of small outbreaks, *s*. Diseases with average transmissibility >*T_c_* will spark large-scale epidemics with probability *S*, which can also be estimated. The value of *T_c_* depends on the contact patterns within a community. Roughly speaking, when abundant opportunities exist for transmission, disease will spread easily, and the epidemic threshold will be low. The equations for *s*, *S*, and *T_c_*, which are entirely in terms of the degree distribution and average transmissibility *T*, are presented in Appendix.

The epidemic potential of disease is commonly estimated by using the basic reproductive number *R*_0_, the number of secondary infections arising from a single infection in a relatively naïve population (*8**,**28*). This quantity is linearly related to the transmissibility of the disease, i.e., *R*_0_ = *γT*, where *γ* depends on the structure of the network (equations 1 and 8 in Appendix). When *T* is at the epidemic threshold (*T* = *T_c_*), then *R*_0_ = 1. Public health interventions aim to reduce the number of new infected cases, ideally decreasing the effective reproductive number of the disease below the epidemic threshold, *R_eff_*<1.

The difference between average transmissibility *T* and the basic reproductive ratio *R*_0_ is important. While both have threshold values that distinguish epidemic from nonepidemic scenarios (*R*_0_ = 1 and *T* = *T_c_*), *T* is determined by the transmission characteristics of the pathogen and the nature of human interactions, but not the numbers of contacts in a community, whereas *R*_0_ depends on all of these factors, particularly on the numbers of interactions within the community. For example, consider a single airborne pathogen spreading through a hospital, where abundant close contacts exist, and through a rural community, where close contacts are rare. The per contact probabilities of transmission (*T_ij_*) may be similar in these settings because they are determined by the pathogenesis of the strain in the host, while the numbers of contacts are different. Therefore, the average transmissibility *T* will be similar in the 2 locations, while *R*_0_ will be substantially higher in the hospital than in the rural setting.

The heterogeneous spread of SARS worldwide suggested context-dependent patterns of transmission with relatively rapid spread through hospitals and relatively slow spread through communities (*29*). A notable exception to this pattern, the large cluster of SARS cases outside a healthcare setting in the Amoy Gardens apartment complex in Hong Kong, seems to have spread through aerosolization of virus-laden sewage rather than direct person-to-person contact (*30*). When contact patterns within a community are extremely heterogeneous, explicitly modeling community structure and *T* makes more sense than assuming a universal *R*_0_. We take this approach to evaluating disease control strategies in an urban setting (*31*).

**Figure 2:**
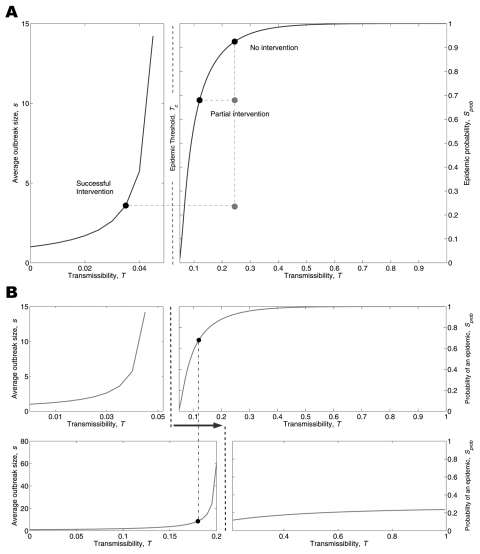
Transmission- vs. contact-reduction intervention. A) Transmission-reduction intervention: solid curves show the average size of an outbreak (left panel) and the probability of a large-scale epidemic (right panel). The horizontal axes cover the spectrum of disease transmissibility (from 0 to 1) such that a single disease is associated with a unique value on either the left curve (if T<Tc) or the right curve (if T>Tc). The epidemic threshold Tc separates the 2 zones. For better visualization, we chose 2 different scales for horizontal axes of the 2 panels. Consider a disease with T = 0.245 (top black circle). A transmission-reduction intervention causes the black circle to slide on a new position on the curve. A successful intervention is the one that lowers T to a value <Tc. B) Contact-reduction intervention: solid curves in the top panel show the epidemiologic vulnerability of the original network. Contact-reduction interventions alter the structure of the contact network and shift the epidemic curves to the right (solid curves in bottom panel). The 2 dashed vertical lines show the critical transmissibility threshold for the old (left) and new (right) networks. Consider the disease denoted by the black circle: the contact-reduction intervention raised the epidemic threshold above transmissibility of the disease and thereby eliminated the possibility of an epidemic.

A primary public health goal is to bring disease from a value above an epidemic threshold to a value below the threshold, thereby eliminating the threat of a large-scale epidemic. This goal can be achieved through interventions that directly affect the transmissibility of the pathogen (*T*) or through interventions that modify patterns of interaction so that the epidemic threshold (*T_c_*) is increased. We call these 2 forms of intervention transmission-reduction and contact-reduction, respectively, and depict them graphically in Figure 2. The solid curves represent the predicted size of an outbreak and the probability of an epidemic for an entire spectrum of *T* from 0 to 1 in an urban setting. All airborne pathogens have a transmissibility value within this range; 0 = no transmission, and 1 = every contact leads to transmission. Thus, any disease can be mapped to a unique value on the curve.

In our simulated urban contact network, the critical transmissibility threshold is *T_c_* = 0.048. An outbreak of disease with *T* = 0.245 will almost certainly spark an epidemic in the absence of intervention (top circle in Figure 2). This value of *T* is equivalent to an *R*_0_ = 5 for this contact network and thus corresponds to a moderately infectious disease like smallpox (*32**,**33*). A successful intervention either reduces *T* so that it lies below *T_c_* (Figure 2A) or modifies the structure of the network so that *T_c_* rises above *T* (Figure 2B). The first strategy can be achieved by interventions that reduce the probability of transmission per contact, such as face masks, gloves, gowns, handwashing, and other infection control precautions that prevent the exchange of respiratory droplets without eliminating contact.

The second strategy involves modifying the contact network itself. Interventions such as quarantine and closing schools and other public places effectively eliminate potential contacts (edges) between persons. Interventions such as immunization and the prophylactic use of antibacterial or antiviral drugs are tantamount to removing persons (vertexes) from the contact network and therefore also alter the network structure. We mathematically assess the effect of such strategies by deleting edges and vertexes from the contact network and predicting the new probability of an epidemic and expected distribution of cases within the community.

## Results

**Figure 3:**
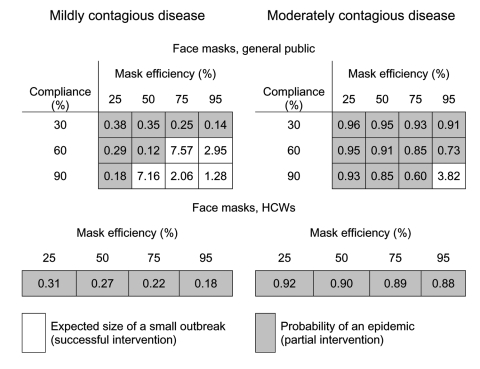
Comparing the effect of face masks for the general public and healthcare workers (HCWs). Mask efficiency is the percent reduction in transmissibility to or from a person correctly using a mask. Compliance is the fraction of the population adopting the intervention. Results are for a mildly contagious disease with a transmissibility T = 0.075 and a moderately contagious disease with a transmissibility T = 0.245. The equivalent basic reproductive number for these diseases are R0 = 1.545 and R0 = 5.047, respectively. Without intervention, both of these diseases have T above the epidemic threshold for the community (Tc = 0.048) and thus may ignite a large-scale epidemic. The probabilities that such epidemics will occur (without intervention) are Sprob = 0.50 and Sprob = 0.97, respectively. Some interventions may not bring T below the epidemic threshold and thus only reduce the probability of an epidemic (gray boxes), while others succeed in containing transmission to a small outbreak (white boxes). Gray boxes give the probability of an epidemic, and white boxes give the expected size of an outbreak. Outbreak size may not be an integer since s is an average taken from all possible outbreaks in the community.

**Figure 4:**
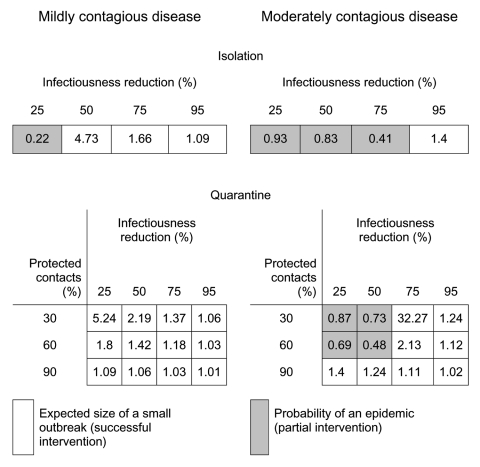
Comparing the effect of isolation and quarantine. Isolation alone reduces the infectious period by a specified percentage. Quarantine involves both isolation and sequestering a fraction of all case contacts. See the Figure 3 caption for further details.

**Figure 5:**
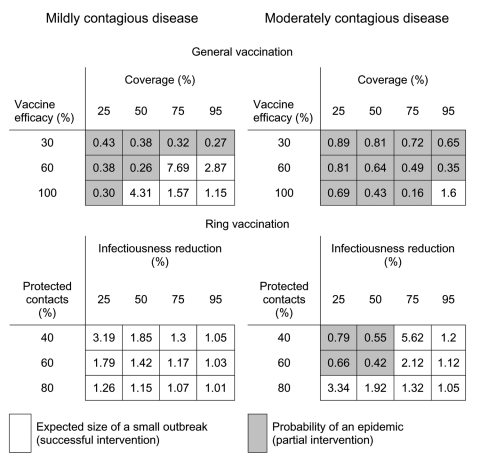
Comparing general vaccination and ring vaccination strategies. General vaccination protects a percentage of persons chosen randomly from the population with an efficacy determined by the vaccine itself. Ring vaccination involves isolating the patient (and the associated reduction in the infectious period) followed by targeted vaccination of contacts. The degree to which contacts are successfully protected depends on the success of contact tracing and the efficacy of the vaccine. See the Figure 3 caption for further details.

We evaluated a variety of commonly implemented public health interventions by changing the contact patterns within the network, transmissibility of the disease, or both. For each strategy, we calculated several epidemiologic quantities: 1) the epidemic threshold, *T_c_*, which may be raised by contact-reduction interventions, 2) the transmissibility of the disease, *T*, which may be reduced by transmission-reduction interventions, 3) if *T*<*T_c_*, the expected size of a small outbreak, *s*, 4) if *T*>*T_c_*, the probability of a large-scale epidemic, *S_prob_*, and 5) if *T*>*T_c_*, the expected size of an epidemic, *S*, should one occur. Based on calculations of these quantities, Figures 3, 4 and 5 report the effect of various interventions applied to a moderately contagious disease just above the epidemic threshold (left panel), where we believe SARS to lie (*34*) and a moderately infectious disease such as smallpox (right panel). Gray entries correspond to unsuccessful interventions; white entries indicate strategies that are predicted to successfully move the pathogen below the epidemic threshold and thereby prevent a large-scale epidemic.

### Transmission Reduction

Although general use of face masks may have a moderate effect, its success hinges on correct use and level of compliance. For instance, face masks that are 75% effective will only prevent a large-scale epidemic of a SARS-like disease if ≥60% of the general population complies perfectly (Figure 3). If persons use face masks incorrectly or only partially, this intervention will be less likely to protect persons and the population as a whole. For moderately contagious diseases like smallpox, face masks alone will not protect large urban areas from an epidemic. Figure 3 also suggests that use of face masks by healthcare workers, while important for personal protection, offers limited protection to the population and does not predictably preclude an epidemic.

One of the factors that influences the transmissibility *T* is the duration of infectiousness. The duration of effective infectiousness may be shortened, but not eliminated, by isolating persons immediately after diagnosis. Although isolating an infected person will physically remove him from the network, the person may already have had a chance to infect others before being identified and isolated. For example, an infectious person who is isolated after the second day of a 6-day infectious period will have had 2 days in which disease could be transmitted to close contacts. Thus, isolation can be effective for diseases with low transmissibility but only if case identification occurs early in the infectious period. For such diseases, an isolation strategy that on average reduces the infectious period by 50% will prevent a large-scale epidemic (Figure 4). Isolation will not preclude an epidemic for a highly transmissible disease unless clinical and diagnostic tools can be applied early and confidently, which may not be the case for an emerging infectious disease.

### Contact Reduction

Contacts between infected and susceptible persons can be eliminated during an outbreak through measures such as quarantine, closing public venues, and ring vaccination, or they can be eliminated preventatively through general vaccination strategies. Figure 4 predicts that simultaneous case-patient isolation and quarantine of close contacts substantially improves containment. For a mildly contagious disease, an outbreak can be controlled with a combination of isolation that reduces the infectious period by 25% and quarantine that successfully sequesters 30% of all case-patient contacts. Much more rigorous isolation and quarantine are required for a highly contagious disease. Such interventions require a strong surveillance infrastructure, reliable rapid diagnostic tests, and social acceptance.

**Figure 6:**
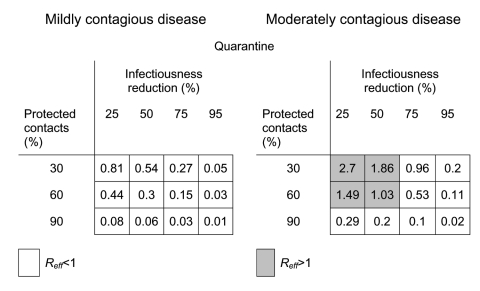
Intervention projections in terms of Reff. This figure presents the results in the lower panel of Figure 4 expressed in terms of effective reproductive number rather than the projected size of an outbreak. If Reff<1 outbreaks will die out, while if Reff>1, epidemics may ensue. Note that the shading indicates epidemic potential and coincides perfectly with the shading in Figure 4.

In Figure 6, we show that such predictions can readily be translated into values of *R_eff_*. Interventions that bring a population under the epidemic threshold are those that decrease *R_eff_* below 1. We emphasize that the predictions in Figures 4 and 6 are specific to the underlying model of contact patterns in an urban setting and that, contrary to common interpretations, *R_eff_* (or *R*_0_) is not a universal constant but instead critically depends on structure of the host community.

### Vaccination

A general vaccination strategy is one in which a substantial proportion of the population is vaccinated at random. The success of this measure depends on proportion (coverage), vaccine efficacy, and disease transmissibility. The availability of a vaccine, therefore, does not guarantee prevention unless both delivery and vaccine-induced immunity are sufficient. For example, Figure 5 shows that a mildly contagious disease like SARS may be thwarted by partial coverage (≈75%) with a moderately efficacious vaccine (≈60% vaccine efficacy). Under this strategy, a moderately contagious disease can become epidemic unless a population receives 95% coverage with a 100% efficacious vaccine.

Ring vaccination of close contacts, on the other hand, is a very effective approach overall. This intervention, like quarantine, involves both transmission and contact reduction. Identifying the index patient results in a reduced infectious period. Subsequent identification and protection of his or her contacts through vaccination further limits the potential spread of the pathogen. Figure 5 considers the effect of ring vaccination on the population as a function of the effectiveness of patient isolation and the fraction of contacts that are successfully immunized. Partial protection of contacts may stem from inadequate contact tracing or an ineffective vaccine. For example, vaccinating 80% of close contacts with a 50% efficacious vaccine is equivalent to vaccinating 40% of close contacts with a 100% efficacious vaccine. Ring vaccination can be a successful strategy for a mildly contagious disease with even a moderate surveillance infrastructure or a partially efficacious vaccine. However, ring vaccination requires more successful case identification, contact tracing, and vaccination when implemented against a highly contagious disease. Ring vaccination is only applicable to diseases with relatively long incubation periods that allow contacts to be identified, vaccinated, and develop a protective immune response. Thus, this strategy is more appropriate for diseases like smallpox (incubation period 12 days) than SARS (incubation period 2–7 days).

### Variation in Outbreak Size

**Figure 7:**
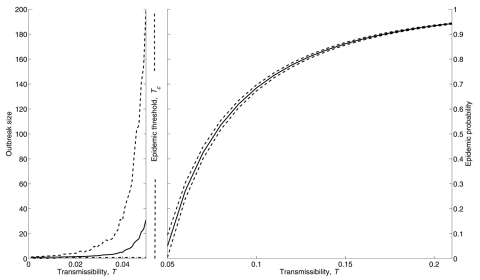
Left panel: variation of outbreak sizes as a function of transmissibility. We generated 1,000 epidemics for each of 20 values of T from 0 to the epidemic threshold. The solid curve represents the mean of outbreak size (m), the dashed curve represents 1 standard deviation above the mean (m + s), and the dotted line at the bottom shows the minimum size of an outbreak, which is always equal to 1, meaning that after the introduction of the first infected case the disease did not spread further. Right panel: sensitivity of epidemic probability to network stochasticity. We generated 100 different networks, each with 2,000 households. Because of the stochastic nature of contact formation during network generation, these 100 networks contain different numbers and configurations of edges and therefore have different degree distributions. The solid curve shows the mean probability of an epidemic across the 100 networks for transmissibilities above the epidemic threshold, and the dashed curves are 95% confidence limits for the mean probability of an epidemic.

The white entries in Figures 3–5 report the expected (average) size of small outbreaks for diseases below the epidemic threshold. Any particular outbreak, however, may not be exactly equal to this average size. In the left panel of Figure 7, we show the average and standard deviation of outbreak sizes over the range of transmissibility values below the epidemic threshold. For each value of *T*, we estimate the standard deviation by using 1,000 simulated epidemics on the original urban network (without intervention). For low *T*, outbreaks tend to be small and close to the average outbreak size *s*. As *T* increases toward the epidemic threshold, the distribution of outbreak sizes widens substantially, and *s* becomes less informative. Given this variability, public health strategies should be based on bringing populations substantially under the epidemic threshold.

### Sensitivity Analysis

Our mathematical predictions are based on a single simulated urban network with 2,000 households with an average of 2.6 people per household. To address the sensitivity of the predictions to the particular pattern of contacts in the network, we stochastically generated 100 urban networks of equal size and predicted the probability of an epidemic for the range of *T* above the epidemic threshold. Since each of these 100 networks has a unique degree distribution, the value of the epidemic threshold varies. In particular we find that the average epidemic threshold is 0.04822 with a 95% confidence interval of 0.04656–0.04988. Recall that the network used in the analysis above has an epidemic threshold *T_c_*=0.048. The right panel of Figure 7 shows the mean probability of an epidemic across these 100 networks with 95% confidence intervals. The probabilities for the particular network that we studied lie very close to the mean probabilities. The narrow confidence intervals suggest that our predictions are fairly robust to the particular architecture of the urban network. We further consider the effect of network size on these predictions in the Appendix.

## Discussion

Using contact network epidemiology, we evaluated various airborne infection control policies for a simulated urban setting like Vancouver. This approach explicitly captures the heterogeneous patterns of interpersonal contacts that lead to disease transmission and allows rapid mathematical prediction of the probability and distribution of an epidemic. This analysis does not depend on computationally intensive simulations. Furthermore, the approach allows one to quantitatively compare strategies that directly reduce the transmissibility of a pathogen or limit opportunities for a pathogen to spread. Although each strategy has been considered on its own, these methods can easily predict the effect of combined interventions for an entire spectrum of airborne infectious diseases, including SARS, smallpox, influenza, and meningococcal meningitis, among others.

Although the qualitative results of this analysis are applied to urban settings, the work is meant to be a proof of concept rather than to provide specific quantitative recommendations for urban control of communicable diseases such as SARS and smallpox. Until we have developed contact network models for a wide range of communities and assessed their generality, contact network epidemiology will need to be applied on a case-by-case basis. For example, hospitals can use these methods to improve control of nosocomial airborne infections. To start, each facility should model its particular network of patient–healthcare worker interactions, then calculate the effect of measures such as respiratory droplet precautions, grouping patients in cohorts, modifications to healthcare worker assignments, and vaccination (12).

The success of contact network epidemiology depends not only on realistic models of contact patterns but also on reliable estimates of the average transmissibility of the pathogen, *T*. As a respiratory pathogen begins to spread through a population, epidemiologists can rapidly identify the mode and rate of disease transmission. These data can provide critical input for intervention strategies. Historically, the rate of disease transmission has been measured and reported in terms of the basic reproductive number *R*_0_, based on the doubling time of case counts in the early phase of an outbreak or epidemic. The value of *R*_0_, however, may vary substantially, depending on the population in which it is measured. For example, recent estimates of *R*_0_ for SARS ranged from 1.2 to 3.6 (34–36). In contrast, *T* is not subject to the particular patterns of interaction within a community and can be reliably estimated in diverse settings. Measuring *T* is only slightly more involved than measuring *R*_0_. For each case, one must measure not just the number of secondary cases, but also the total number of contacts of the case-patient during the infectious period and then divide the first value by the second.

Just as enormous molecular and technological resources are often mobilized to develop vaccines and diagnostic tools for emerging infectious diseases of public health importance, we should also harness the powerful quantitative mathematical tools that help assess disease interventions. When an airborne pathogen strikes, public health officials should be able to make scientifically grounded decisions about the competing medical, economic, and social implications following deployment of control measures. We illustrate that contact network epidemiology can provide detailed and valuable insight into the fate and control of an outbreak. Integrating these tools into public health decision making should facilitate more rational strategies to manage emerging diseases, bioterrorist events, and pandemic influenza in situations in which empiric data are not yet available to guide decision making.

## Appendix

### Materials and Methods 1: Simulating Urban Center Networks

**Figure A1:**
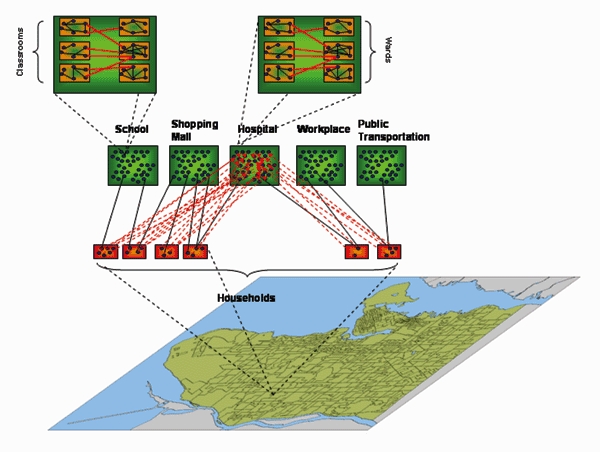
Schematic diagram of an urban network. We used the demographic data for the Greater Vancouver Regional District to build the contact network.

**Figure A2:**
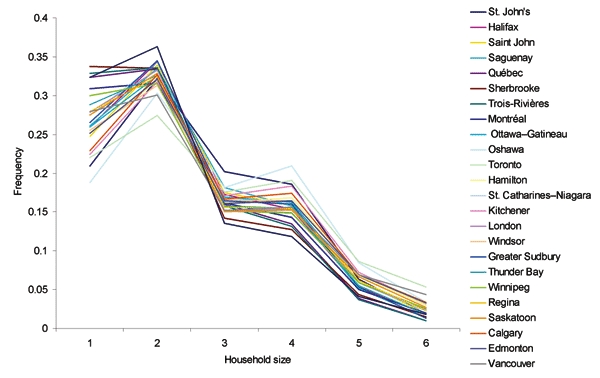
Household size distribution in Vancouver, Toronto, and other Canadian metropolitan areas. The data presented here are publicly available online at the Statistics Canada Web site from http://www.statcan.ca/english/Pgdb/famil53e.htm

The simulated urban center network that we used in our analysis is based on the demographic data for the Greater Vancouver Regional District (GVRD), British Columbia, Canada, with a population of 2 million people. The data that we use in our simulations are publicly available at Web sites for Statistics Canada (http://www.statcan.ca), BC Statistics (http://www.bcstats.gov.bc.ca), the Centre for Health Services and Policy Research at the University of British Columbia (http://www.chspr.ubc.ca), the city of Vancouver (http://www.city.vancouver.bc.ca), and the Vancouver Public School Board (http://www.vsb.bc.ca/default.htm). According to Statistics Canada, Vancouver has 758,715 households and an average household size of 2.6 with a distribution as shown in Figure A1. Qualitatively similar distributions of household sizes occur in 24 other cities across Canada with population sizes from 120,000 to 4,600,000. We begin assembling an urban-centered network (Figure A1) by generating a predetermined number of households with a size distribution that corresponds to Figure A2. We then classify persons in each household as children, adults, or seniors, according to Vancouver demographics. Next, on the basis of age, we assign persons to schools, work, hospitals, and other public places. Finally, we determine the contact patterns among persons that share households or go to the same public places.

**Figure A3:**
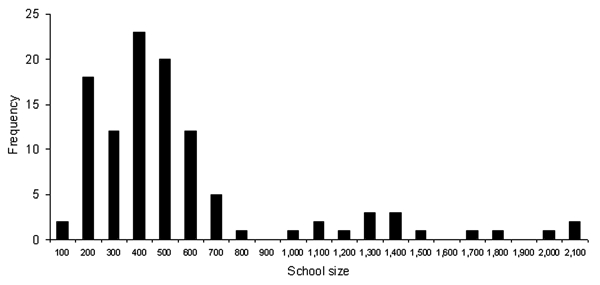
School size distribution for Vancouver. The data presented here are in the Vancouver School Board December 2002 Ready Reference, publicly available online from http://www.vsb.bc.ca/board/publications.htm

The number and sizes of public places (schools, hospitals, shopping malls, workplaces, and generic public spaces) are largely based on publicly available statistics for Vancouver. For example, we assign students to schools on the basis of the distribution of elementary and secondary school sizes in Vancouver, which range from 100 to 2,100 students (Figure A3). All children are assigned to a school, minus a fraction corresponding to the population <5 years of age. All adults go to work, minus a fraction that corresponds to the Vancouver unemployment rate. Seniors do not go to work. People are assigned to hospitals in proportion to reported occupancy of Vancouver hospital beds. At this point, the assumed distributions of shopping centers and generic public spaces are based more on intuition than on statistics. Finally we assume occasional contacts between households that represent visitations of neighbors and friends.

To make contacts between persons within these spaces, we typically assume a specified probability that 2 persons in the same place are in contact with each other. This probability ranges from 1 in the case of persons within the same household to very low values for persons who visit the same shopping center. This results in Poisson distributions of contact rates within specific settings.

Each household is a completely connected small network; that is, every person in a household is connected to every other person in that household. Since infection transmission occurs with probability *T*<1, a single infected member of a household will not necessarily transmit the disease to all members of the household. To make contacts within schools, we first randomly divide students into subsets corresponding to classrooms, with average size 25. We then create contacts between any 2 students in the same classroom with a specified probability 0.3 and between any 2 students in different classrooms within the same school at a lower probability. Within each school is also a group of adults (representing staff) that has high rates of contact across classrooms. Each hospital is similarly divided into wards, with patients primarily contacting others within their ward and caregivers with high rates of contact across multiple wards. These edges, which are based on typical rates of hospital bed occupancy, reflect hospital-based activity unrelated to the outbreak. In addition, we connect each person to a subset of healthcare workers in their local hospital by directed edges, thereby capturing hospital-based contacts that occur only if and when a person becomes infected by the pathogen in question. If a typical person does not become infected, he will not visit the hospital and thus not come in contact with healthcare workers. Except for this class of conditional contacts, all edges in the network are undirected, which indicates that transmission can take place in either direction between the 2 persons who are in contact with each other. Within workplaces, shopping malls, and general public spaces, we assume very low probabilities of contact between persons (0.003–0.030).

**Figure A4:**
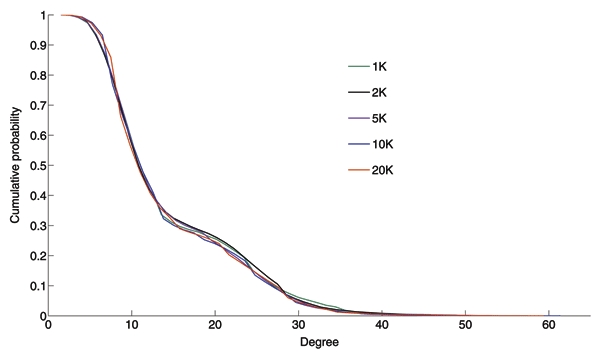
The cumulative undirected-degree distributions for urban networks with 1,000, 2,000, 5,000, 10,000, and 20,000 households corresponding to population sizes 2,595, 5,337, 13,080, 25,722, and 51,590 persons.

**Figure A5:**
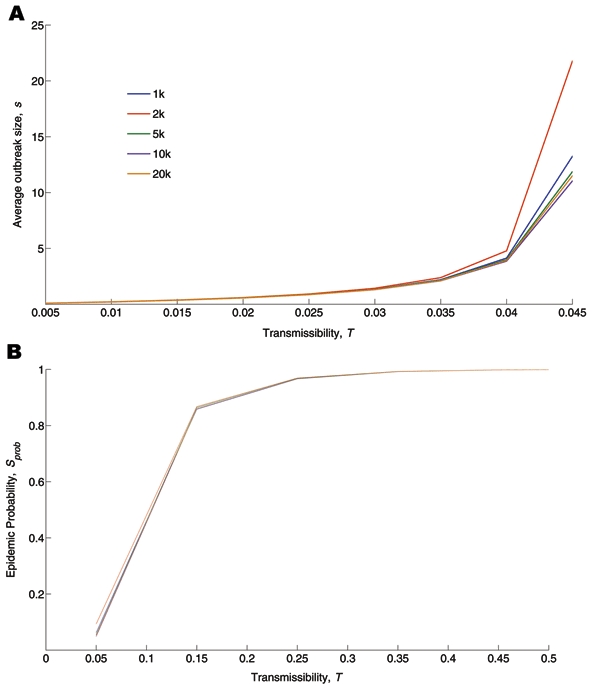
Average size of small outbreaks (top) and the epidemic probability (bottom) for 5 different networks introduced in Figure A4.

For the purposes of this analysis, we simulated an urban network with 2,000 households (≈5,200 persons). We used this relatively small network because it permitted extensive epidemic simulation as a means to verify our analytical predictions. Indeed, we found that the simulations agree well with the analysis (37). We considered the sensitivity of our results to the size of the network. Increasing the size of the network does not have a profound effect on the results as long as the criteria for making social contacts remain the same. In particular, we found that as the network size increases from 1,000 households to 20,000 households, the probability of an epidemic remains almost identical, and the average size of a small outbreak varies slightly. Figure A4 shows the undirected-degree distributions of 5 networks with 1,000, 2,000, 5,000, 10,000, and 20,000 households (corresponding to population sizes of 2,595, 5,337, 13,080, 25,722, and 51,590 persons). The in-degree and out-degree distributions (not shown) are likewise very similar among the 5 networks. Despite a 20-fold increase in the size of network, the distributions vary only slightly. Figure A5 shows that the epidemic probabilities predicted for these networks are virtually indistinguishable, while the average outbreak size varies slightly.

**Figure A6:**
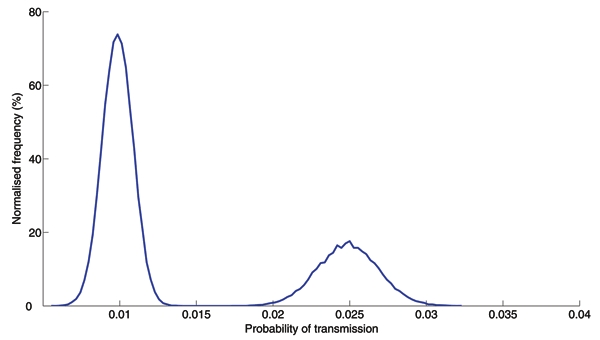
Transmission probability distribution. The probability of transmission of respiratory pathogens depends on the amount of shedding, distance, duration of contact, and environmental factors such as temperature and humidity. Reports on SARS epidemiology suggest a bimodal distribution of transmission probabilities: close contacts in hospitals may have had high probabilities of transmission while typical contacts in schools, workplaces, and shopping malls may have had low probabilities of transmission (2,3). We assumed this distribution of per day transmission probabilities across the entire network in our analysis. The first peak corresponds to household contacts, while the second peak (with higher probability of transmission) corresponds to all contacts occurring in healthcare settings. Note that this distribution includes the per day probabilities of transmission for all possible contacts in the network, and thus has a smaller mean than the estimates reported for SARS in which the probability of transmission was estimated for particular settings (2,3).

The transmissibility value assumed for a mildly contagious disease (*T* = 0.075) in Figures 3–5 in the main text was based on the following analysis. If a person *i* is infectious for a period of *τ* days, the transmissibility of infection between persons *i* and *j*, *T_ij_*, is 1 – (1 – *p_ij_*)*^τ^_i_*, where *p_ij_* is the probability of transmission (per day). Since the duration of infectiousness and symptoms vary from person to person, *T_ij_* will not be identical for all edges in the network. We assumed a Gaussian distribution with mean 6 (days) and standard deviation 0.4 for the values of *τ* throughout the network (38). Figure A6 gives the distribution of the *p_ij_*, the per day probabilities of transmission. Both distributions are based on information about transmission of severe acute respiratory syndrome in the absence of intervention (38,39). The transmissibility assumed for a moderately contagious disease (*T* = 0.245) was selected because it is equivalent to an average reproductive ratio of *R*_0_ ≈ 5. For this case, we assumed that all contacts in the graph shared this average transmissibility.

For both diseases, we model intervention strategies by decreasing the duration of infectiousness, the probability of transmission for specific contacts in the network, or both. We then calculate the average transmissibility *T* of the modified network for use in percolation analysis, as reported in Figures 3–5 in the main text.

### Materials and Methods 2: Epidemic Analysis

In a mixed undirected-directed network (henceforth semidirected network), each vertex (person) has an undirected degree representing the number of undirected edges joining the vertex to other vertexes as well as both an in-degree and an out-degree representing the number of directed edges coming from other persons and going to other persons, respectively. The undirected degree and in-degree indicate how many contacts can spread disease to the person and thus are related to the likelihood that a person will become infected during an epidemic; the undirected degree and out-degree indicate how many contacts may be infected by that person should he become infected; thus, they are related to the likelihood that a person will ignite an epidemic.

Given the degree distribution of the contact network, one can analytically predict the fate of an outbreak. Let *p_jkm_* be the probability that any given person in the population has in-degree = *j*, out-degree = *k*, and undirected-degree = *m*, and let *T* be the average transmissibility of the disease, that is, the probability that transmission of the disease occurs between an infected person and a susceptible person that are in contact with each other.

In the main text, we report the following quantities: the basic reproductive number *R*_0_, the epidemic threshold *T_c_*, the average size of an outbreak *s*, and the probability that an epidemic will occur *S_prob_*. Newman previously derived these quantities for a completely undirected network (40). Here we provide the corresponding formulas for a semidirected network. We derive these quantities by using percolation theory and generating function methods in another manuscript (37,41).

The basic reproductive number can be written as

(1)
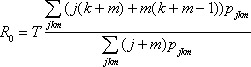
.

The expression for the transmissibility threshold value is

(2)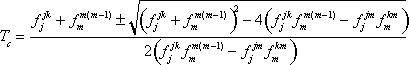,

where


(3)

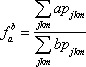



and therefore 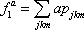.

The average size of an outbreak is given by

(4)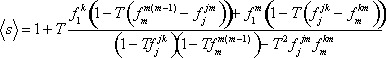,

and the probability of an epidemic is given by the following expression:

(5)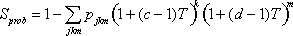,

where *c* and *d* are the solutions to the self-consistency equations


(6)

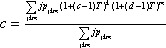



and

(7)
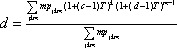
.

We use numerical root finding methods to solve for *c* and *d*.

For a completely undirected network equation (1) can be simplified as

(8)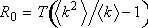,

where *k* is the degree of the persons leading to an undirected degree distribution, *p_k_* (37,40). If the degrees follow a Poisson distribution with an average degree of *C*, then *k* = *C* and *k*^2^ = *C*(*C* + 1) and *R*_0_ = *CT*. In epidemiologic compartmental models, this equation has often been used as *R*_0_ = *CpD*, where *C* is the average contact rate, *p* is the average probability of transmission, and *D* is the average duration of infectiousness. Therefore, the results from compartmental models only describe an undirected contact network with a Poisson distribution. The degree distribution of a real-life urban network may vary substantially from this assumption (37).

## Footnotes

2 For the purposes of this manuscript, "airborne" refers to respiratory pathogens that are spread through respiratory secretions and can be either airborne, such as tuberculosis, or dropletborne, such as SARS.
